# Should Prophylactic Endotracheal Intubation Be Performed in Upper Gastrointestinal Bleeding?

**DOI:** 10.7759/cureus.64567

**Published:** 2024-07-15

**Authors:** Syed Bilal Pasha, Zahid Ijaz Tarar, Harleen Chela, Aidan McDermott, Jocelyn Ihnat, Michelle L Matteson-Kome, Yezaz A Ghouri, Matthew L Bechtold

**Affiliations:** 1 Gastroenterology and Hepatology, University of Missouri, Columbia, USA; 2 Internal Medicine, University of Missouri, Columbia, USA; 3 Internal Medicine, Harry S. Truman Memorial Veterans’ Hospital, Columbia, USA; 4 Internal Medicine/Gastroenterology and Hepatology, University of Missouri School of Medicine, Columbia, USA; 5 Gastroenterology, University of Missouri, Columbia, USA

**Keywords:** length of stay, pneumonia, meta-analysis, bleeding, prophylactic intubation

## Abstract

No consensus exists on the standard of intraoperative airway management approach to prevent endoscopy complications in acute gastrointestinal (GI) bleeding. Eight years after our initial meta-analysis, we reassessed the effect of prophylactic endotracheal intubation in acute GI bleeding in hospitalized patients. Multiple databases were reviewed in 2024, identifying 10 studies that compared prophylactic endotracheal intubation (PEI) versus no intubation in acute upper GI bleeding in hospitalized patients. Outcomes of interest included pneumonia, length of hospital stay, aspiration, and mortality. The odds ratio (OR) or mean difference (MD) using the random effects model was calculated for each outcome. In total, 11 studies (10 retrospective, one prospective) were included in the meta-analysis (n = 7,332). PEI demonstrated statistically significant higher odds of pneumonia (OR = 5.83; 95% confidence interval (CI) = 3.15-10.79; p < 0.01) and longer length of stays (MD = 0.84; 95% CI = 0.12-1.56; p = 0.02). However, mortality (OR = 1.68; 95% CI = 0.78-3.64; p = 0.19) and aspiration (OR = 2.79; 95% CI = 0.89-8.7; p = 0.08) were not statistically significant. PEI before esophagogastroduodenoscopy for hospitalized upper GI bleeding patients is associated with an increased incidence of pneumonia within 48 hours and prolonged hospitalization but no statistically significant increased risk of mortality or aspiration.

## Introduction and background

Upper gastrointestinal bleeding (UGIB) is a prevalent and serious global health issue, resulting in around 350,000 hospital admissions annually in the United States alone [1]. Peptic ulcer disease (PUD) and variceal bleeding are among the leading causes of UGIB. Despite advances in medicine, UGIB continues to be linked with high mortality rates, reaching up to 10% and 20% for variceal and non-variceal bleeding cases, respectively [2]. Esophagogastroduodenoscopy (EGD) is currently the standard diagnostic and therapeutic tool for managing UGIB [3].

Although EGD is generally considered safe in the average-risk population, with low complication and mortality rates, the risk may be altered in the setting of UGIB. Cardiopulmonary complications are of particular concern, accounting for a significant proportion of all adverse events and fatalities. Intraoperative aspiration of blood and gastric contents is thought to be a frequent cause of these complications [4,5].

Endoscopists and intensivists often recommend prophylactic endotracheal intubation (PEI) to avert the triggering aspiration event. However, critically ill patients with UGIB are complex and have the potential for hemodynamic instability, rendering this practice contentious [3]. The lack of supporting evidence remains the most significant obstacle to the safe, efficient, and effective management of such patients [6]. Bearing this in mind, our objective was to perform a meta-analysis of the available data to address the controversy of PEI in patients with UGIB.

## Review

Methodology

Data Search and Screening

A comprehensive electronic literature search of Medline/OVID, PubMed, Scopus, Google Scholar, and Web of Science databases was performed in January 2024. This meta-analysis was conducted in accordance with the Preferred Reporting Items for Systematic Review and Meta-Analysis (PRISMA) statement. Search terms included [(endotracheal intubation or intubation or prophylactic) AND (endoscopy or EGD or esophagogastroduodenoscopy) AND (UGIB or gastrointestinal bleeding or upper GI bleeding) AND (adult)]. A manual search of the bibliographies of the included articles was performed (JI, MMK, and MLB) to identify any studies that may have been missed during the initial literature search.

Study Selection

Study selection was performed by numerous reviewers (SP, ZIT, IJ, MMK, and MLB). The authors independently screened the abstracts, titles, and full manuscripts to identify the studies eligible for inclusion. English-language studies of adult patients (age >18 years) describing the effect of PEI before EGD in hospitalized GI bleeding patients were included. Outcomes of pneumonia, hospital length of stay, aspiration, and mortality were examined.

Data Extraction

Data was extracted by two independent reviewers (ZT and UF). The authors extracted the following data from identified studies: Study design, cohort characteristics, rate of adverse events of pneumonia, hospital length of stay, aspiration, and mortality. The authors reviewed the extracted data sheet and prepared the final datasheet.

Quality Assessment

We evaluated the quality of included non-randomized studies using the Cochrane Risk of Bias Tool for Non-randomized Studies (ROBINS-I) [7]. Two authors (ZT and UF) conducted the quality assessment separately, and any disagreement was resolved by consensus with a third reviewer (MB).

Statistical Analysis

RevMan 5.4 (Review Manager, Version 5.4, Copenhagen: The Nordic Cochrane Centre, The Cochrane Collaboration, 2012) was utilized for statistical analysis. The authors calculated the mean difference (MD) and corresponding 95% confidence interval (CI) for continuous outcomes and pooled odds ratio (OR) with corresponding 95% CI for dichotomous outcomes. Random effects model was used to calculate the pooled OR with 95% CI and a p-value <0.05 was deemed statistically significant. The I^2^ statistics and Cochran’s Q test were used for heterogeneity and variance. Publication bias was assessed by funnel plots.

Results

Literature Search

**Figure 1:**
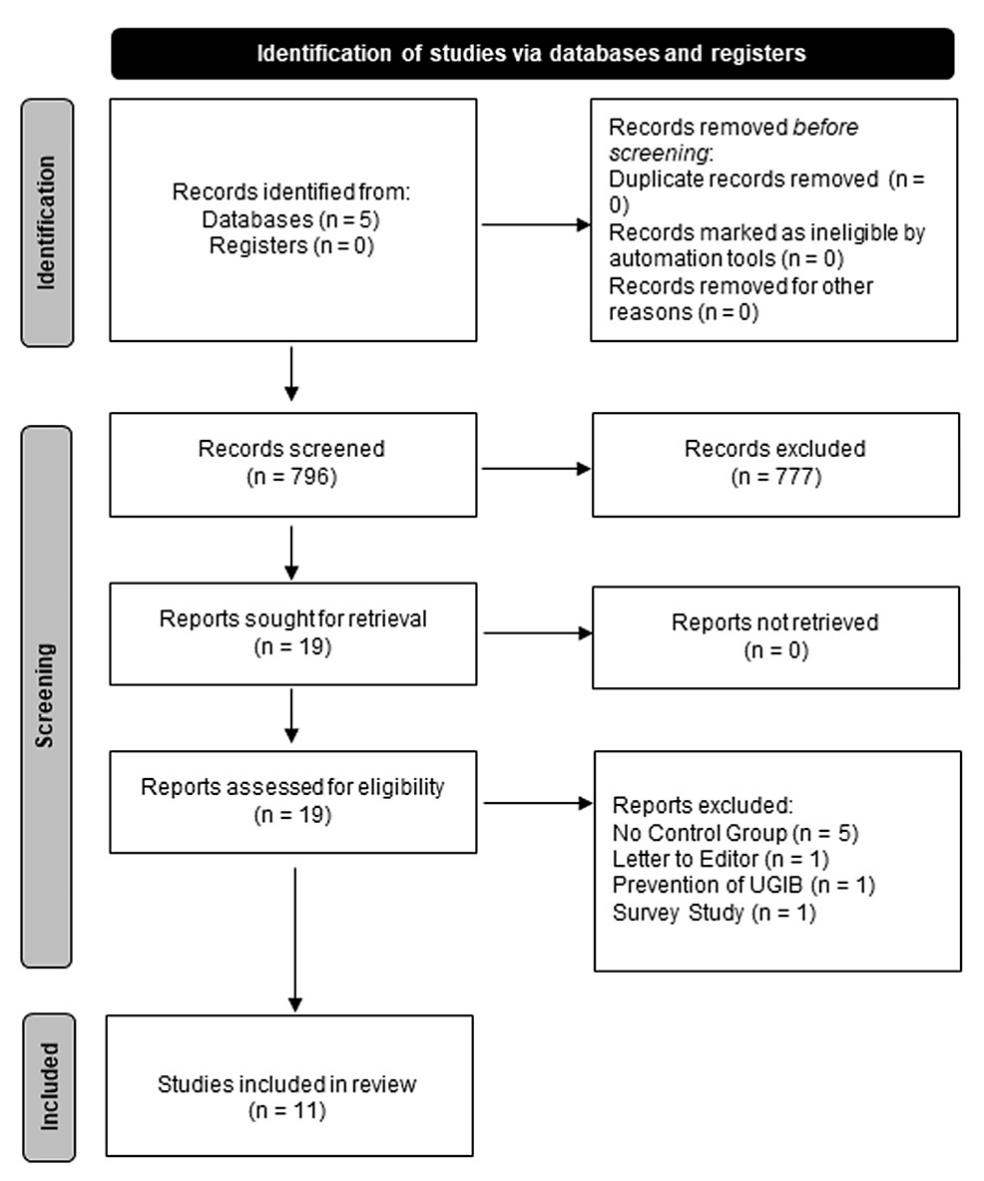
Literature search flowchart.

The initial electronic search identified 796 potentially relevant studies. The following inclusion criteria were considered: prophylactic endotracheal intubation, UGIB, English language, adult patients (age >18 years), and the presence of a control arm. We excluded 785 studies based on lack of inclusion criteria or missing data; thus, 11 studies were included in the meta-analysis (Figure 1).

Of the 11 included studies, four [8-11] were abstracts, and seven were manuscripts [2-5,12-14] (Table 1).

**Table 1:** Details of the studies included in the meta-analysis. UGIB = upper gastrointestinal bleeding; PEI = prophylactic endotracheal intubation

Study	Study type	Location	Study duration	# of patients (n)	Demographics	UGIB source	PEI (n)	No PEI (n)
Koch et al. [4] 2006	Retrospective	Alabama	1995–2002	62	Male: 44, Female: 18, Mean age: 48–49	Variceal	42	20
Rehman et al. [13] 2009	Retrospective	Minnesota	2002–2006	98	Male: 61, Female: 37, Median age: 62–68	All	49	49
Tang et al. [5] 2017	Retrospective	Nevada	2008–2013	110	Male: 75, Female: 35, Mean age: 53–56	Variceal	65	45
Hayat et al. [2] 2017	Retrospective	Ohio	2011–2014	200	Male: 127, Female: 73, Mean age: 59	All	100	100
Lohse et al. [3] 2015	Prospective	Denmark	2006–2013	3,580	Male: NA, Female: NA, Mean age: NA	PUD	2,101	1,479
Perisetti et al. [12] 2019	Retrospective	Arkansas, Texas	2000–2013	89	Male: NA, Female: NA, Mean age: NA	All	69	20
Lin et al. [14] 2023	Retrospective	China	2014–2015	946	Male: 557, Female: 389, Mean age: NA	All	108	838
Abdulsamad et al. [8] 2016	Retrospective	New York	2008–2014	1,474	Male NA, Female NA, Mean Age NA	All	264	1,210
Lee et al. [9] 2016	Retrospective	Ohio	2004–2012	156	Male: NA, Female: NA, Mean age: NA	All	78	78
Stipho et al. [10] 2006	Retrospective	Arizona	NA	151	Male: NA, Female: NA, Mean age: NA	All	15	136
Suchartlikitwong et al. [11] 2020	Retrospective	Texas	2012–2017	466	Male: NA, Female: NA, Mean age: NA	All	22	444

The study design consisted of one prospective cohort study [3] and 10 retrospective cohort studies. Other than one study from Denmark [3] and one study from China [14], the remainder of the publications were from the United States. The study populations consisted of all types of UGIB [2,8-14], cirrhosis/variceal bleeding [4,5], and PUD [3]. The data collection period ranged from 1954 to 2017 [11]. Outcomes of interest were pneumonia within 48 hours, hospital length of stay, aspiration, and mortality.

Aspiration Events

**Figure 2:**
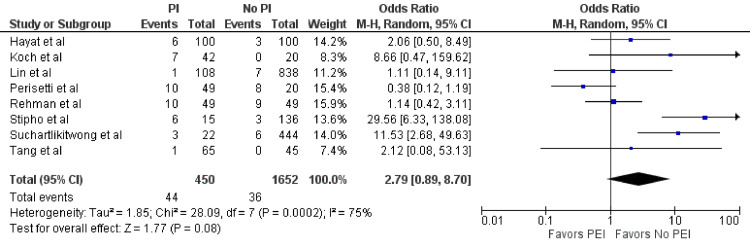
Aspiration. Forest plot demonstrating the comparison of prophylactic intubation versus no intubation for patients with UGIB for aspiration [2,4,5,10-14]. UGIB = upper gastrointestinal bleeding; PEI = prophylactic endotracheal intubation

Eight studies (n = 2,102) examined aspiration events in both groups [2,4,5,10-14]. In the PEI group, 44 out of 450 (9.8%) patients experienced an aspiration event compared to 36 out of 1,652 patients (2.2%) in the no-PEI group. Despite a trend favoring fewer odds of aspiration in the no-PEI group, this difference was not statistically significant (OR = 2.79; 95% CI = 0.89-8.7; p = 0.08) (Figure 2). However, significant heterogeneity was observed (I^2^ = 75%, p < 0.01).

Pneumonia Within 48 Hours

**Figure 3:**
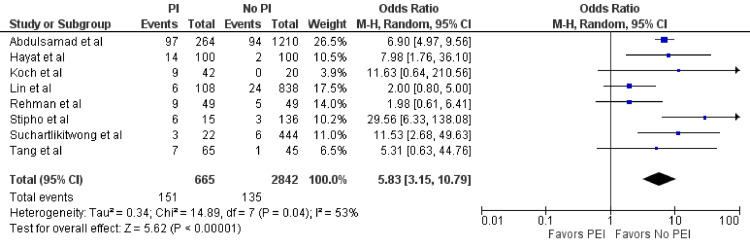
Pneumonia. Forest plot demonstrating the comparison of prophylactic intubation versus no intubation for patients with UGIB for pneumonia within 48 hours [2,4,5,8,10,11,13,14]. UGIB = upper gastrointestinal bleeding; PEI = prophylactic endotracheal intubation

Eight studies (n = 3,507) assessed the occurrence of pneumonia within 48 hours [2,4,5,8-11,13,14]. Among the 665 patients who underwent PEI before EGD for UGIB, 151 (22.7%) developed pneumonia within 48 hours. In comparison, 135 out of 2,842 patients (4.8%) in the group without PEI experienced pneumonia within the same time frame. This difference was statistically significant (OR = 5.83; 95% CI = 3.15-10.79; p < 0.01) (Figure 3). Additionally, significant heterogeneity was detected (I^2^ = 53%, p = 0.04).

Mortality

**Figure 4:**
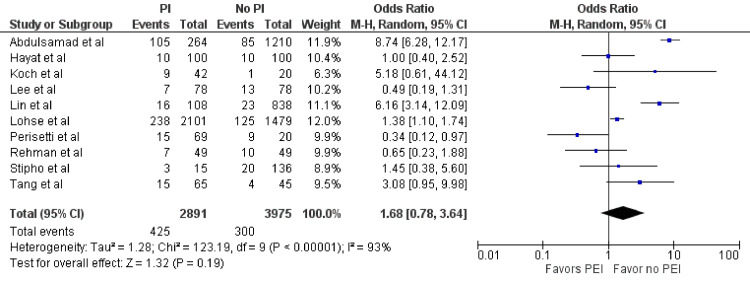
Mortality. Forest plot demonstrating the comparison of prophylactic intubation versus no intubation for patients with UGIB for mortality [2-5,8-10,12-14]. UGIB = upper gastrointestinal bleeding; PEI = prophylactic endotracheal intubation

Ten studies included in our meta-analysis assessed mortality (n = 6,866) [2-5,8-10,12-14]. Among the PEI group, mortality was observed in 425 out of 2,891 patients (14.7%), while 300 out of 3,975 patients (7.5%) experienced mortality in the no-PEI group. No statistically significant difference was found between the two groups (OR = 1.68; 95% CI = 0.78-3.64; p = 0.19) (Figure 4). However, significant heterogeneity was observed (I^2^ = 93%, p < 0.01).

Length of Hospital Stay

**Figure 5:**
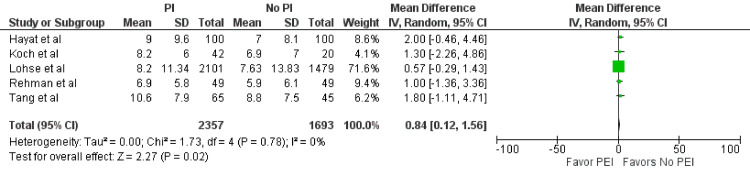
Length of hospital stay. Forest plot demonstrating the comparison of prophylactic intubation versus no intubation for patients with UGIB for the length of hospital stay [2-5,13]. UGIB = upper gastrointestinal bleeding; PEI = prophylactic endotracheal intubation

Nine studies [2-5,8,10-13] evaluated the total length of hospital stay in days, but due to incomplete data, only five studies (n = 4,050 days) were included in the analysis [2-5,13]. The combined length of hospital stay was 2,357 days for the PEI group and 1,693 days for the no-PEI group. The PEI group experienced a significantly longer length of stay (MD = 0.84; 95% CI = 0.12-1.56; p = 0.02) (Figure 5). No statistically significant heterogeneity was detected (I^2^ = 0%, p = 0.78).

Publication Bias

**Figure 6:**
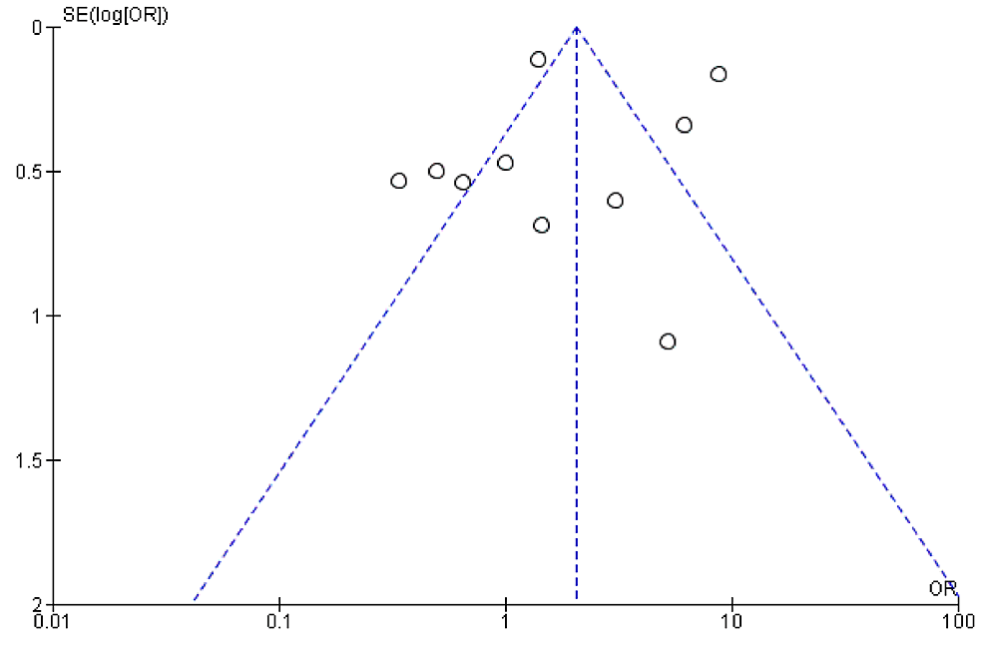
Funnel plot. Funnel plot showing no publication bias [2-5,8-10,12-14].

No significant publication bias was noted by funnel plots (Figure 6).

Quality Assessment

The quality of the studies was assessed using ROBINS-I [7] (Table 2).

**Table 2:** Quality assessment. Quality assessment using the Cochrane Risk of Bias Tool for Non-randomized Studies (ROBINS-I) [2-5,7,8-14].

Study	Study type	Bias due to confounding	Bias in the selection of participants in the study	Bias in the classification of interventions	Bias due to deviations from intended interventions	Bias due to missing data	Bias in the measurement of outcomes	Bias in the selection of reported result	Overall risk of bias
Koch et al. [4] 2006	Retrospective	Moderate	Moderate	Low	Low	Low	Low	Low	Moderate
Rehman et al. [13] 2009	Retrospective	Moderate	Moderate	Low	Low	Low	Low	Low	Moderate
Tang et al. [5] 2017	Retrospective	Serious	Moderate	Low	Low	Low	Low	Low	Moderate
Hayat et al. [2] 2017	Retrospective	Serious	Moderate	Low	Low	Low	Moderate	Moderate	Serious
Lohse et al. [3] 2015	Prospective	Moderate	Low	Low	Low	Low	Low	Low	Low
Perisetti et al. [12] 2019	Retrospective	Moderate	Moderate	Low	Low	Low	Moderate	Moderate	Moderate
Lin et al. [14] 2023	Retrospective	Moderate	Moderate	Low	Low	Low	Low	Low	Moderate
Abdulsamad et al. [8] 2016	Retrospective	Moderate	Moderate	Low	Low	Moderate	Low	Moderate	Moderate
Lee et al. [9] 2016	Retrospective	Moderate	Moderate	Low	Low	Low	Low	Low	Moderate
Stipho et al. [10] 2006	Retrospective	Serious	Serious	Moderate	Low	Moderate	Low	Low	Serious
Suchartlikitwong et al. [11] 2020	Retrospective	Moderate	Moderate	Low	Low	Low	Low	Low	Moderate

​​​​​Given the retrospective nature of nearly all studies, significant bias is apparent. Lohse et al. was the only study found to have a low overall risk of bias [3]. Overall, the risk of bias was moderate in seven studies [4,5,8,9,11-13] and serious in two studies [2,10].

Discussion

Although endoscopy is a relatively safe procedure, it has been associated with several adverse events, including cardiopulmonary complications such as aspiration and pneumonia. The overall complication rates for EGD range from 0.6 to 5.4 per 1,000, but in cases of UGIB, this can increase to up to 12-22% [15]. Some studies have suggested that emergency intubation can lead to complications such as esophageal intubation, development of new pulmonary infiltrates, and even death within 30 minutes of intubation, particularly in hemodynamically unstable patients [16]. Emergency endoscopy for UGIB carries a more than 10-fold increase in complication rates (8%) when compared to non-emergency endoscopy (0.7%). Cardiopulmonary complications account for 23-50% of all these adverse events and are responsible for 50-60% of fatalities [5]. Aspiration during endoscopy and the resulting aspiration pneumonia have become significant issues, prompting a rise in the utilization of PEI before EGD, even though there is scarce evidence to suggest any improvement in patient outcomes [5].

Previous retrospective studies of PEI in UGIB have shown varying results regarding aspiration, pneumonia, and mortality rates [4,5,12,13]. To evaluate these outcomes, a meta-analysis of four studies comparing UGIB patients who received PEI to those who did not was performed in 2015 [6]. The meta-analysis demonstrated a significantly higher incidence of pneumonia within 48 hours in the prophylactically intubated group, while trends toward worse outcomes for aspiration and mortality were noted, but were not statistically significant [6].

The common rationale for PEI is airway protection, particularly in the setting of brisk UGIB, where patients are typically intubated for airway protection when the endoscopy is deemed risky to conduct without a secure airway [5,13]. However, indications and thresholds for PEI vary among physicians. Studies have indicated that only a minority of gastroenterologists believe that patients with unstable vital signs or ongoing hematemesis should be prophylactically intubated [17]. Rehman et al. found similar baseline clinical features between the intubated and non-intubated groups, and the decision to perform endotracheal intubation appeared to have been made subjectively and was largely dependent on the endoscopist’s preference [13]. Generally, younger patients who are sicker and have massive GI bleed presenting as hematemesis are more likely to undergo PEI [2,11,12].

There also appears to be a temporal trend in the practice of PEI. Its use for EGD has become more prevalent in recent times. Rudolph et al. discovered that in their institution, significantly fewer patients received intubation before EGD in 1988 compared to 1992, even though the overall proportion of intubations during hospitalization remained unchanged [15]. Similarly, Koch et al. found that newer faculty were more inclined to the practice of PEI [4]. These findings align with data from Tang et al., where most UGIB cases between 2008 and 2013 involved PEI [5].

The suspected etiology of GI bleed also plays a role in the severity and risk of complications, in turn influencing the decision for PEI. Acute variceal hemorrhage (AVH) is known to present with more severe bleeding and a large volume of blood in the upper GI tract. Consequently, AVH poses a high risk of aspiration from emergent EGD which may contribute to cardiopulmonary complications [4,5]. This has been reported to occur at a rate of 2.4-3.3% [4]. Concurrent hepatic encephalopathy (HE) from decompensated cirrhosis can worsen a patient’s ability to protect their airway, increasing the risk of aspiration [5]. Perisetti et al. and Hayat et al. indicated that PEI was more commonly performed in patients with cirrhosis or a history of alcohol abuse, who had a higher likelihood of variceal bleeding [2,12].

Of the 11 studies included in our analysis, two [4,5] specifically examined PEI in variceal bleeders (VBs), while Lohse et al. focused only on non-VB cases [3]. The remaining studies included both types of bleeders or did not specify [2,8-13]. Tang et al. found no differences in rates of aspiration, pneumonia, and hospital length of stay in prophylactically intubated VBs, but post-EGD intensive care unit stay was longer in this group [5]. Koch et al., on the other hand, found higher rates of aspiration events in intubated VBs (19% vs. 0%; p = 0.01) but no significant difference in mortality or hospital length of stay [4]. Although both studies only included VBs, there were subtle differences in their inclusion criteria. Koch et al. [4] only included patients with grade 1 HE, while Tang et al. included patients with Grade 1 and 2 HE, the latter of which were more commonly seen in the PEI group (21.5 vs. 8.9%; p = 0.117) [5]. In contrast, Lohse et al. included patients undergoing emergent EGD for PUD. Their results indicate that younger patients with more severe presentation and excessive alcohol intake were more likely to undergo PEI. They found increased in-hospital mortality in the PEI group (11.3% vs. 8.5%; p = 0.005), but this was non-significant after adjustment (OR = 1.18; p = 0.188). The length of hospital stay was similar between PEI and no intubation groups (p = 1.108) [3].

Peri-procedural aspiration events remain a common trigger for cardiopulmonary complications seen with EGD and are thus a major reason behind the decision for PEI. A study from Australia that utilized a radiotracer to identify aspiration during routine non-urgent EGD for different indications than UGIB failed to identify any meaningful aspiration, establishing the safety of the procedure [18]. However, in the setting of UGIB, an increased risk of cardiopulmonary complications, including aspiration, is often observed. In this regard, studies comparing pre- and post-EGD chest radiographs (CXRs) have shown the development of new infiltrates suggesting intraoperative aspiration events in 14-20% of cases [15]. In reality, this could be even higher owing to the lack of sensitivity of CXR in detecting micro-aspiration. Seven studies included in our analysis examined aspiration events in the PEI and no-intubation groups [2,4,5,10-13]. The results of our meta-analysis do not show a statistically significant improvement in aspiration events with PEI; in fact, it shows a trend toward higher rates of aspiration associated with this practice. However, the results of these studies should be interpreted with caution because, except for one, all are of retrospective design and are thus prone to confounding biases and show considerable heterogeneity as well.

Pneumonia is another concern related to EGD procedures, and it is often considered when deciding on PEI. Seven studies included in our analysis investigated the relationship between PEI and pneumonia rates [2,4,5,10-13]. Our meta-analysis revealed no statistically significant reduction in pneumonia rates with the use of PEI. However, as with aspiration events, the results should be interpreted cautiously due to the retrospective design of most studies, which can lead to confounding biases and considerable heterogeneity.

The impact of PEI on mortality and hospital length of stay is another important consideration. Some studies in our analysis reported higher mortality in the PEI group [3], while others found no significant difference [4]. Our meta-analysis did not show a significant improvement in mortality rates with PEI. Similarly, there was no significant difference in length of stay between the PEI and no-intubation groups. These findings should also be interpreted with caution due to the retrospective design of the studies and the potential for confounding biases and significant heterogeneity.

## Conclusions

Our meta-analysis of the available literature on PEI before EGD for UGIB suggests that PEI does not significantly improve patient outcomes in terms of reducing aspiration events, pneumonia rates, mortality, or hospital length of stay. On the contrary, PEI may be associated with higher odds of pneumonia within 48 hours and longer length of stay. However, these findings should be interpreted cautiously due to the retrospective design of most studies and the potential for confounding biases and heterogeneity among them. Further prospective, randomized controlled trials are needed to establish definitive evidence on the use of PEI in the context of EGD for UGIB.

## References

[1] Kamboj AK, Hoversten P, Leggett CL (2019). Upper gastrointestinal bleeding: etiologies and management. Mayo Clin Proc.

[2] Hayat U, Lee PJ, Ullah H, Sarvepalli S, Lopez R, Vargo JJ (2017). Association of prophylactic endotracheal intubation in critically ill patients with upper GI bleeding and cardiopulmonary unplanned events. Gastrointest Endosc.

[3] Lohse N, Lundstrøm LH, Vestergaard TR, Risom M, Rosenstock SJ, Foss NB, Møller MH (2015). Anaesthesia care with and without tracheal intubation during emergency endoscopy for peptic ulcer bleeding: a population-based cohort study. Br J Anaesth.

[4] Koch DG, Arguedas MR, Fallon MB (2007). Risk of aspiration pneumonia in suspected variceal hemorrhage: the value of prophylactic endotracheal intubation prior to endoscopy. Dig Dis Sci.

[5] Tang YM, Wang W (2017). Prophylactic endotracheal intubation prior to urgent endoscopy in patients with suspected variceal hemorrhage: an evaluation of outcomes and complications. J Gastroenterol Hepatol Res.

[6] Almashhrawi AA, Rahman R, Jersak ST (2015). Prophylactic tracheal intubation for upper GI bleeding: a meta-analysis. World J Metaanal.

[7] Sterne JA, Hernán MA, Reeves BC (2016). ROBINS-I: a tool for assessing risk of bias in non-randomised studies of interventions. BMJ.

[8] Abdulsamad M, Kamireddy C, Karki N (2016). Should we intubate the patient first? Outcomes of prophylactic endotracheal intubation for upper gastrointestinal bleeding. Am J Gastroenterol.

[9] Lee PJ, Hayat U, Ullah H, Lopez R, Vargo JJ (2016). Prophylactic endotracheal intubation in critically ill patients with upper gastrointestinal bleeding is associated with higher cardiopulmonary unplanned events. Gastrointest Endosc.

[10] Stipho S, Ramirez FC (2006). The clinical outcomes of electively intubated patients undergoing urgent/emergent EGD for acute UGI bleeding. Am J Gastroenterol.

[11] Suchartlikitwong S, Juarez RM, Jaroudi S (2020). Elective endotracheal intubation prior to EGD for upper gastrointestinal bleeding increased risk of cardiopulmonary events. Gastroenterology.

[12] Perisetti A, Kopel J, Shredi A, Raghavapuram S, Tharian B, Nugent K (2019). Prophylactic pre-esophagogastroduodenoscopy tracheal intubation in patients with upper gastrointestinal bleeding. Proc (Bayl Univ Med Cent).

[13] Rehman A, Iscimen R, Yilmaz M (2009). Prophylactic endotracheal intubation in critically ill patients undergoing endoscopy for upper GI hemorrhage. Gastrointest Endosc.

[14] Lin Y, Song F, Zeng W (2023). Cardiopulmonary prognosis of prophylactic endotracheal intubation in patients with upper gastrointestinal bleeding undergoing endoscopy. World J Emerg Med.

[15] Rudolph SJ, Landsverk BK, Freeman ML (2003). Endotracheal intubation for airway protection during endoscopy for severe upper GI hemorrhage. Gastrointest Endosc.

[16] Schwartz DE, Matthay MA, Cohen NH (1995). Death and other complications of emergency airway management in critically ill adults. A prospective investigation of 297 tracheal intubations. Anesthesiology.

[17] Waye JD (2000). Intubation and sedation in patients who have emergency upper GI endoscopy for GI bleeding. Gastrointest Endosc.

[18] Thomson A, Tye-Din J, Tonga S, Scott J, McLaren C, Pavli P, Lomas F (2007). Aspiration in the context of upper gastrointestinal endoscopy. Can J Gastroenterol.

